# Performance of Graphite and Titanium as Cathode Electrode Materials on Poultry Slaughterhouse Wastewater Treatment

**DOI:** 10.3390/ma13204489

**Published:** 2020-10-10

**Authors:** Kulyash Meiramkulova, Davud Devrishov, Nurbiy Marzanov, Saida Marzanova, Aliya Kydyrbekova, Tatyana Uryumtseva, Lyazzat Tastanova, Timoth Mkilima

**Affiliations:** 1 Department of Environmental Engineering and Management, Faculty of Natural Sciences, L.N. Gumilyov Eurasian National University, Satpayev Street 2, Nur-Sultan 010000, Kazakhstan; kuleke@gmail.com (K.M.); aliyafromkz@gmail.com (A.K.); 2 Department of Immunology and Biotechnology, Moscow State Academy of Veterinary Medicine and Biotechnology, 23 Scryabin Street, Moscow 109472, Russian; davud@mgavm.ru (D.D.); adiga.m@mail.ru (S.M.); 3 Laboratory of molecular basis of breeding, L.K.Ernst Federal Science Center for Animal Husbandry, Dubrovitsy 60, Podolsk Municipal District, Moscow Region 142132, Russia; nmarzanov@yandex.ru; 4 Department of Agriculture and Bioresources, Innovative University of Eurasia, Lomov Street 45, Pavlodar 14008, Kazakhstan; vbh2@mail.ru; 5 Department of Chemistry and Technology, K.Zhubanov, Aktobe Regional State University, A.Moldagulova Avenue 34, Aktobe 030000, Kazakhstan; lyazzatt@mail.ru; 6 Department of Civil Engineering, Faculty of Architecture and Construction, L.N. Gumilyov Eurasian National University, Satpayev Street 2, Nur-Sultan 010000, Kazakhstan

**Keywords:** electrode material, aluminium, graphite, titanium, poultry slaughterhouse, wastewater treatment

## Abstract

Despite the potential applicability of the combination between aluminium (anode) and graphite or titanium (cathode) for poultry slaughterhouse wastewater treatment, their technical and economic feasibilities have not been comprehensively captured. In this study, aluminium (anode) and graphite and titanium as cathode electrode materials were investigated and compared in terms of their performance on poultry slaughterhouse wastewater treatment. The wastewater samples collected from the Izhevsk Production Corporative (PC) poultry farm in Kazakhstan were treated using a lab-based electrochemical treatment plant and then analyzed after every 20 and 40 min of the treatment processes. Cost analysis for both electrode combinations was also performed. From the analysis results, the aluminium–graphite electrode combination achieved high removal efficiency from turbidity, color, nitrite, phosphates, and chemical oxygen demand, with removal efficiency ranging from 72% to 98% after 20 min, as well as 88% to 100% after 40 min. A similar phenomenon was also observed from the aluminium–titanium electrode combination, with high removal efficiency achieved from turbidity, color, total suspended solids, nitrite, phosphates, and chemical oxygen demand, ranging from 81% to 100% after 20 min as well as from 91% to 100% after 40 min. This means the treatment performances for both aluminium–graphite and aluminium–titanium electrode combinations were highly affected by the contact time. The general performance in terms of removal efficiency indicates that the aluminium–titanium electrode combination outperformed the aluminium–graphite electrode combination. However, the inert character of the graphite electrode led to a positive impact on the total operating cost. Therefore, the aluminium–graphite electrode combination was observed to be cheaper than the aluminium–titanium electrode combination in terms of the operating cost.

## 1. Introduction

Excessive usage of water in the poultry slaughterhouse production processes is also proportional to the release of huge amounts of wastewater. The estimated amount of wastewater generated from a poultry production process ranges from 20 to 40 L per bird, with 25 L being a typical value [1]. Moreover, the generated wastewater is characterized by high pollution strength, which includes parameters such as high biochemical oxygen demand (BOD), chemical oxygen demand (COD), high suspended solids (TSS), and a complex mixture of fats, proteins, and fibers requiring systematic treatment before discharge or recycling [2]. The efficiency of the treatment process is, however, highly dependent on the type of technology used. In general, poultry slaughterhouse wastewater (PSWW) can be treated by using physical, chemical, and biological treatment systems. Each treatment technology possesses advantages and disadvantages. Physical treatment processes such as membrane filtration systems are among the most highly efficient treatment systems in terms of pollutant removal, but they are pressure-demanding and the generated sludge has to be separately handled, making the processes relatively expensive and associated with feasibility issues for large scale treatments [3]. The biological treatment systems (anaerobic and aerobic) are characterized by the high adaptability of microorganisms to a wide variety of wastewater composition. However, the biological treatment systems are slow processes requiring large physical areas and generating large amounts of sludge [4]. Chemical-based treatment systems such as electrochemical (EC) processes provide an alternative PSWW treatment, with several advantages such as being robust, requiring small space, being easy to operate, and having flexibility under fluctuating wastewater composition [5]. Different electrode materials may, however, perform differently even from wastewater with similar characteristics, which makes it challenging to predict the behavior of electrochemical wastewater treatment systems [6]. EC treatment methods are among the most widely used technologies for the treatment of different types of wastewater, including; textile [7,8], saline wastewater [9], olive mill wastewater [10], tannery wastewater [11,12,13], as well as pharmaceuticals [14].

In the recent past, there has also been a growing interest in EC technologies for PSWW treatment [15,16]. The EC wastewater treatment approach can be accomplished in many different ways, including direct oxidation and reduction reactions, as well as through the generation of reactive chemical species, or by releasing chemicals that achieve physical removal of the pollutants in water [17]. Some of the typical examples of EC water treatment technologies are EC oxidation used for mineralization of organic pollutants [18], EC-based water disinfection [19], removal of cyanides [20], and EC reduction used for metals recovery and the conversion of highly toxic and persistent organic compounds to less toxic forms [21]. In general, the EC wastewater treatment approach is characterized by high adaptability, relatively high energy competence, acquiescence to automation as well as environmental friendliness; it is perceived to be an advanced technology used for the treatment of PSWW [22]. According to Zarei et al. [23], up to 100%, microbial removal efficiency was achieved when copper electrodes were used for PSWW disinfection.

An EC system consists of at least two electrodes; an anode and a cathode as well as an intermediate space filled with electrolyte [24]. The definition of anode and cathode is more dependent on the direction of current passing through the electrode than the voltage polarity of electrodes. Therefore, an anode can be defined as an electrode through which conventional current (positive charge) flows into the device from the external circuit, while a cathode is an electrode through which conventional current flows out of the device [25]. This means if by any chance the direction of the current through the electrodes is reversed, with a typical example of a rechargeable battery when it is subjected to a charging process, the naming of the electrodes defining the anode and cathode is also reversed. Either a voltage source (electrolysis cell) or an electrical load (galvanic element) is essential for the electrical circuit, which is closed through electrical wires [5]. There are many electrode materials used for EC treatment technologies including aluminum, graphite (Gr), and titanium (Ti), as well as iron in a plate or packed form of scraps such as steel turnings and millings [26,27]. Being electronically conductive, and then being able to interact with the molecule are among the basic features of electrode materials [28]. Gr is among the electrodes that are considered inert because when they are utilized in the EC treatment systems, they do not participate in any chemical reactions whatsoever. The inert electrode materials are neither consumed nor added to [29]. Therefore, unlike Gr, the aluminum and Ti electrodes have the potential to be consumed during the EC treatment process.

When the EC system is connected to a power source, the oxidation process occurs in the anode making it electrochemically corroded, while passivation occurs in the cathode [30]. Moreover, the EC process contains the production of coagulants in situ by electrically dissolving metal electrodes [31]. The metal ion production occurs at the anode, while hydrogen gas from the electrolysis of water is produced at the cathode. The metal hydroxide ions adsorb and trap contaminants while the hydrogen gas floats the particles present in water [32].

Taking an example of aluminium (Al) and iron (Fe) electrodes, their general processes in the EC system can be summarized using chemical Equations (1) and (2) [33].

At anode electrode surface:

(1) $$M\;\to M_{\left(aq\right)}^{3+}+3e^{-}$$

At cathode electrode surface:

(2) $$3H_{2}O+3e^{-}\to \frac{3}{2}H_{2}+3OH^{-}$$

where *M* represents Al or iron electrode.

The generated $M_{\left(aq\right)}^{3+}$ and *OH^−^* ions react to form various hydroxo monomeric and polymeric species, depending on the pH range, which transforms finally into M(OH)_3_ according to complex precipitation kinetics [34]. Finally, the soluble and colloidal pollutants are adsorbed by the coagulants and then removed by either sedimentation or flotation.

However, the performance of electrochemical methods for PSWW treatment can be highly dependent on the type of electrode material used [35], characteristics of the wastewater, as well as the scale at which the particular treatment plant is operating [36]. In addition, the operating cost of an EC treatment system is highly determined by the energy consumed within the system and the cost of material, mostly because of electrode consumption [37]. The combination of Al and iron electrodes is among the most widely used electrode material combinations for PSWW treatment [38,39]. Despite aluminium electrode material being characterized by low cost, easy availability, and relatively less oxidation and toxicity [40], as well as the potential of Gr as an inert electrode material and the usability of Ti, their combinations have been not comprehensively captured previously for PSWW treatment. Information about the technical and economic feasibility of combining the Al electrode (anode) with Gr or Ti as cathode for PSWW treatment is still scarce. It should also be noted that, depending on the characteristics of wastewater to be treated, the application of Al as an anode material can be a significant challenge [41]. Therefore, it is of great importance to investigate the applicability of Al material for a specific type of wastewater before its application due to the possibility of forming dense alumina film on the electrode surface when used as an anode material [42]. Moreover, when the wastewater subjected to an EC treatment system comes from different sources within a poultry farm, it is also important to observe the effect of mixing ratios on the general performance of the system [43]. The present study focuses on the performance of Gr and Ti electrode materials when used as cathode electrodes, with Al as the anode electrode, on PSWW purification.

In this study, Gr and Ti electrode materials are comparatively analyzed to investigate their performance when combined with aluminum (anode) electrode material for PSWW treatment. The wastewater samples were collected from the slaughterhouse of the Izhevsk Production Corporative (PC) poultry farm located in Izhevsk village, Arshalinsky district, in Akmola region about 70 km from the capital city Nur-Sultan in Kazakhstan and then treated using the lab-based electrochemical treatment plant. The electrode materials are investigated in terms of their removal efficiency for physicochemical parameters and operating costs. The effect of contact time, based on 20 and 40 min increments of the treatment processes, is also investigated.

## 2. Materials and Methods

### 2.1. Case Study, Water Samples, and Analytical Methods

The water samples were collected from the Izhevsk PC poultry slaughterhouse located in Izhevsk village, Arshalinsky district, in Akmola region of Kazakhstan, about 70 km from the capital city Nur-Sultan (51°10′ North latitude and 71°26′ East longitude). Samples were collected as grab samples before and after treatment using 5 L plastic bottles, which were thoroughly rinsed with deionized water before use. All samples were preserved at 4 °C before analysis. Eleven water quality parameters were investigated during the Al–Gr experiment, namely, pH, turbidity, color, TSS, free chlorine, total chlorine, nitrite, nitrate, phosphates, ammonium, and COD. The list was extended to seventeen parameters including total iron, manganese, nickel, chromium, aluminum, and BOD when the wastewater was subjected to the Al–Ti electrodes combination. 

Furthermore, a number of scientific procedures and tools were used to assess the state of water quality. The concentrations of COD, free chlorine, total chlorine, nitrites, nitrates, total phosphorous, and ammonium before and after the treatment were determined using the combination of a spectrophotometer (Hach DR3900, HACH/LANGE, Berlin, Germany) and colorimeter (Hach DR900, Berlin, Germany), with standard reagents as well as the test kits. The US Environmental Protection Agency Great Lakes National Program Office (GLNPO), Washington, D.C., Standard Operating Procedure for Turbidity was used for the measurements of turbidity [44]. The American Public Health Association (APHA) 4500-Nor was used to determine the concentrations of total phosphorous in the samples [45], while the lab pH-meter (Hach Co), Frederick, MD, US, was used for pH measurements. Determination of color concentration in the samples was achieved using the UV-V Spectrophotometer (PE-5400UV) pr-in ECOCHEMICAL, St. Petersburg, Russia [46], and the TSS was measured using the Hach TSS portable hand-held turbidity meter Hach Co, HACH/LANGE, Berlin, Germany. In general, analyses of all the studied samples were accomplished following the recommendations in the APHA Standard Methods for the Examination of Water and Wastewater [47]. The general characteristics of the PSWW are presented in Table 1, in terms of minimum concentration values (Min), maximum values (Max), arithmetic mean (AM) as well as standard deviation (SD). 

### 2.2. Experimental Setup

**Figure 1:**
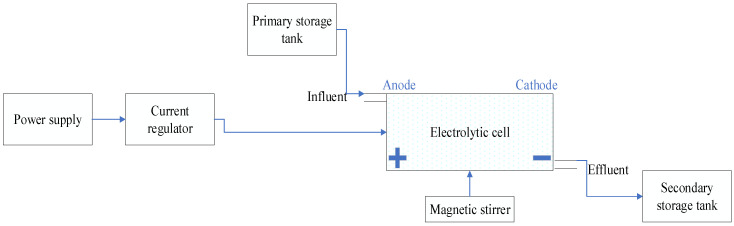
Experimental flowchart.

The quantity of wastewater used was 1.7 L for each session of the experiments. The general setup of the EC treatment system includes a reactor of 15 × 13 × 11 cm^3^ dimension made of polypropylene material in which the electrodes were placed. Direct current was supplied to both electrodes (potentiostatic mode), with design power supply (Xinhua Electrical Weld Company, Loudi City, China) ranging from 0 to 50 V for voltage and 0 to 10 A for current density. Aluminum was used as an anode electrode with dimensions of 10.8 × 11.8 × 0.2 cm^3^, while Gr and Ti were used as cathode electrodes with dimensions of 10.8 × 11.8 × 0.7 cm^3^. Both configurations had the same fixed distance of 2 cm between electrodes and were placed parallel to the reactor. The current density varied with the electrode material and wastewater characteristics. The general settings are summarized in Table 2 and Figure 1.

The effect of contact time was also investigated, of which samples were collected and analyzed after every 20 and 40 min of the treatment processes.

### 2.3. Statistical Analysis

After the analysis of raw wastewater and the purified water, the results were subjected to statistical analysis, which included determination of removal efficiencies from the studied electrode materials. The removal efficiencies were presented in terms of percentages for better visualization of the performance from the studied the treatment approaches with respect to the studied electrode materials. The approach used for the treatment efficiency computations is summarized in Equation (3).

(3) $$T_{e}\left(\%\right)=\frac{C_{b}-C_{a}}{C_{b}}\times 100$$

where *T_e_* is treatment efficiency, *C_b_* is the initial concentration from raw wastewater, and *C_a_* is the concentration of a parameter.

Correlation analysis was performed for some of the physicochemical parameters to evaluate the strength of the relationship among the selected parameters. This means a high correlation indicates that two or more variables have a strong relationship with each other; thus, a case of weak correlation means that the variables are hardly related. The interpretation of the correlation coefficients used in this study is summarized in Table 3.

Furthermore, box and whisker plots were also used for water quality data analysis. The plots visually showed the distribution of numerical data and skewness through displaying the data quartiles (percentiles) and averages. From the perspective of statistical analysis, box plots show the five-number summary of a set of data: including the minimum score, first (lower) quartile, median, third (upper) quartile, and maximum score. In this study, turbidity, COD, TSS, and color were selected for statistical analysis using box and whisker plots for the Al–Gr and Al–Ti electrode combinations.

Estimation of the operational costs for the studied electrodes was based on the current unit prices in Kazakhstan, Equations (4)–(6). The electricity price in the market is 0.05 US$/kWh, the Al electrode average price is 32.25 US$/kg, Ti is 50 US$/kg and that of the Gr electrode is 16.5 US$/kg. It should also be noted that Gr is a stable inert electrode material, which means that it is not consumed during the electrochemical process, leading to a positive impact on the total operating cost as no replacement is required.

The computation of the operating cost from the energy and electrode consumption was achieved through the monitoring of the energy consumed at the end of each experiment (after 40 min) as well as quantification of the electrode materials used during the entire study.

(4) $$Operating\;cost=X\times Energy_{consumpion}+Y\times Electrode_{consumption}$$

where *X* is the price of particular electrode material per kg based on the Kazakhstani Market in 2020, *Y* is a unit price for electricity in Kazakhstan.

The amount of energy consumed by the electrodes during the EC treatment processes per each cubic meter of water was estimated using Equation (5).

(5) $$Energy_{consumption}=\frac{\left(V\times I\times t\right)}{v}$$

where *V* is voltage, *I* is current (Ampere), *t* is EC system operating time (in seconds) and *v* is the volume of the treated wastewater (m^3^). 

Moreover, Faraday’s law of electrolysis was used for the estimation of the electrode material consumption, as illustrated in Equation (6):

(6) $$Electrode_{consumption}=\frac{I\times t\times M_{w}}{z\times F\times v}$$

where *M*_w_ is the molar mass of the electrode element (26.98 g/mol), *F* is Faraday’s constant (96,485 C/mol), and *z* is the number of electrons transferred.

## 3. Results and Discussion

### 3.1. Water Quality Analysis Results

The analysis of physicochemical parameters from the raw wastewater as well as treated effluent samples subjected to the Al–Gr and Al–Ti electrodes’ combinations was accomplished. From raw wastewater, the average concentration values for turbidity and color were 218.88 FAU and 1999.17°, respectively, nitrite and nitrate were 0.09 mg/L and 18.35 mg/L, respectively, while phosphates, ammonium, TSS, and COD were 4.84 mg/L, 1.33 mg/L, 403.33 mg/L, and 2096.67 mg/L, respectively. However, from Table 4, it can be observed that the treatment processes under the Al–Gr electrodes combination achieved 0 mg/L as the minimum recorded concentration value for several parameters including turbidity, TSS, nitrite, nitrate, and COD, which is equivalent to 100% removal efficiency. From the average concentrations, 1.25 FAU was achieved from turbidity after the treatment processes using the Al–Gr electrode combination, which is equivalent to 99.4% removal efficiency. From color, 88° was achieved as an average concentration value, equivalent to 95.6% removal efficiency. From TSS, 7.17 mg/L was achieved as an average concentration value, equivalent to 98.2% removal efficiency. For the rest of the parameters (free and total chlorine, phosphates, ammonium, and COD), removal efficiencies ranging from 18.7% to 94.2% were achieved, with the lowest removal efficiency observed from ammonium. In the literature, other EC treatment methods for PSWW were also observed to have high performance for parameters such as turbidity, color, TSS, and COD was also observed in some other studies [48]. According to Kanjana Chanworrawoota and Mali Hunsom [49], high removal efficiencies of TSS, color, and COD were also observed from pulp and paper mill industry wastewater using EC treatment methods. 

From Table 5, a high correlation between turbidity and color is observed, with a correlation index of 0.908926. In the literature, color and turbidity have also been to be linearly correlated [50], as turbidity concentrations in water can be affected by the colored dissolved organic matter [51]. Apart from turbidity and color, turbidity and TSS are also observed to be highly correlated with a correlation index of 0.907. According to Hannouche et al. [52], the study confirmed the existence of a strong linear relationship between turbidity and TSS within a combined sewer system. Moreover, following the high correlation between turbidity and TSS, Oliveira et al. [53], suggested that measurement of turbidity can be a faster and economically efficient option for estimating TSS.

A high correlation is also observed between COD and other parameters such as turbidity, color, and TSS, with a correlation index ranging from 0.922 to 0.990. In general, COD is an important indicator of water quality, which can be defined as a measure of the amount of oxygen being consumed during the oxidation process of oxidizable organic matter in the presence of a strong oxidizing agent; it is generally used to indirectly quantify the amount of organic matter in water [54], of which the presence of turbidity, color, and TSS in water is a result of suspended and dissolved organic matter. 

**Figure 2:**
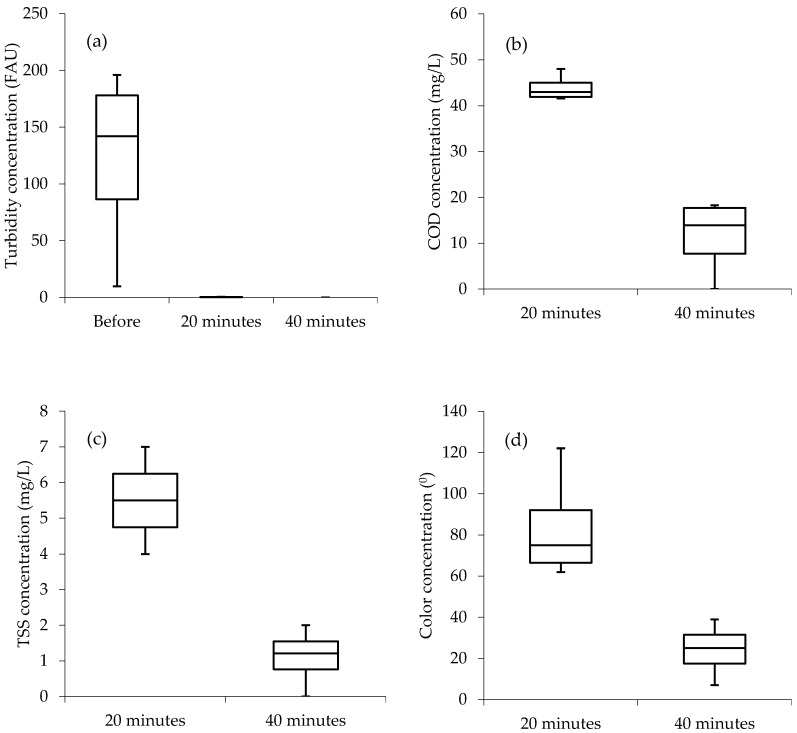
Boxplots (Al–Gr); (**a**) turbidity, (**b**) COD, (**c**) TSS, (**d**) color; Before = before treatment.

Figure 2 provides a summary of the water quality analysis results before treatment as well as after 20 and 40 min of the EC treatment processes from turbidity, COD, TSS, and color using the Al–Gr electrode combination. The box and whisker plots reveal further the significant influence of the contact time on the wastewater purification performance of the Al–Gr electrode combination. The portray further the high efficiency of the Al–Gr electrode combination on the removal of turbidity, COD, TSS, and color in the PSWW. The boxplots from Figure 2a,b (40 min) show the median closer to the upper or top quartile; for that matter, the distribution of water quality data from the studied parameters is considered to be “negatively skewed”. This means the data constitute a higher frequency of more low concentration values than the high concentration values within the list of the studied samples. While from Figure 2b (20 min) and Figure 2d (20 min), the median is observed to be closer to the lower quartile meaning that the water quality data constitute a higher frequency of more high concentration values than the low concentration values (“positively skewed”). Moreover, in the boxplots from Figure 2c (20 and 40 min) and Figure 2d (40 min), the median is observed to be closer to the middle, indicating that the water quality data distribution is symmetric or normal. 

Furthermore, from the boxplots, it can be observed that the Al–Gr electrode combination achieved 100% removal efficiency from turbidity within 20 to 40 min of contact time. However, there is a high chance that if more time was to be applied after 40 min, then the treatment process could have achieved 100% removal efficiency from COD, TSS, and color.

From Table 6, it can be observed that the combination of Al and Ti electrodes achieved 0 mg/L as the minimum recorded concentration value for a number of parameters including turbidity, color, TSS, nitrite, and ammonium, which is 100% removal efficiency. However, the Al–Ti combination was able to achieve 0 mg/L as a minimum concentration value from ammonium, which was not achieved when the wastewater was subjected to the Al–Gr combination.

From the average concentrations, 0 FAU was achieved from turbidity after treatment using the Al–Ti electrode combination, which is equivalent to 100% removal efficiency. From color, 29° was achieved as an average concentration value, equivalent to 98.5% removal efficiency. From TSS, 2 mg/L was achieved as an average concentration value, equivalent to 99.5% removal efficiency. In addition, BOD and COD were among the parameters that were associated with high removal efficiency from the Al–Ti electrode combination. From the average concentrations, 98.6% removal efficiency was achieved from BOD, as well as 94% from COD.

For the rest of the parameters (free and total chlorine, phosphates, ammonium, nitrite, nitrate, total iron, manganese, nickel, chromium, and aluminum), the removal efficiencies ranged from 42.9% to 92.5%, with the lowest removal efficiency observed from ammonium.

**Figure 3:**
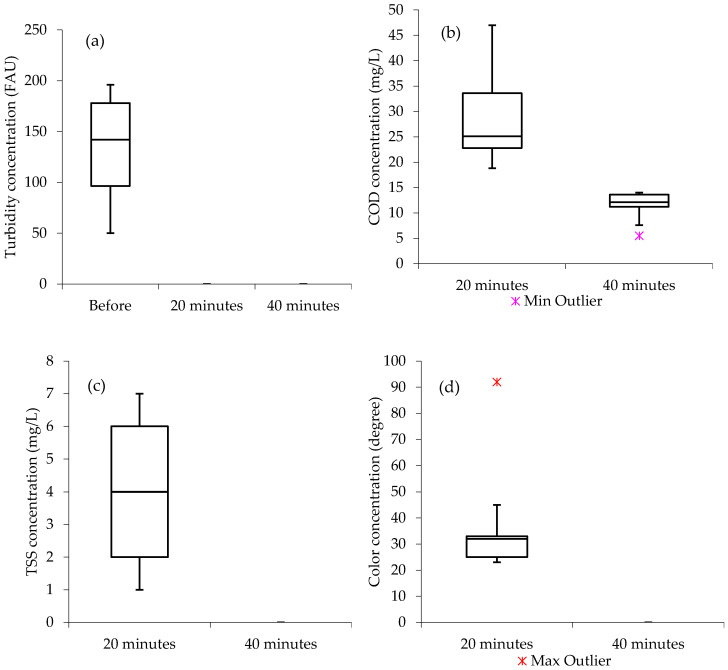
Boxplots (Al–Ti); (**a**) turbidity, (**b**) COD, (**c**) TSS, (**d**) color; Before = before treatment; Min = minimum; Max = maximum.

Figure 3 presents the box and whisker plots as a summary of the water quality analysis results before treatment as well as after 20 and 40 min of the treatment processes from turbidity, COD, TSS, and color using the Al and Ti electrodes combination. From Figure 3a, it can be observed that there is a significant difference between the concentrations of the turbidity before treatment and the ones after treatment (20 and 40 min), of which the Al–Ti electrodes combination achieved 100% turbidity removal efficiency for both 20 and 40 min. In addition, from the COD boxplots (Figure 3b), it can be observed that the median from both 20 and 40 min water quality analysis results are closer to the lower quartile; the COD data distribution from Al–Ti electrode combination was “positively skewed”, meaning that the data constitute a higher frequency of more high COD concentrations than the low valued concentrations within the list of the studied samples, while the color analysis results in Figure 3d show that the data distribution was “negatively skewed”, with the median closer to the upper quartile. This means, within the list of the studied samples, there was a higher frequency of more low color concentrations than the high valued concentrations. In general, the color concentrations after the 20 min of treatment processes ranged between slightly above 20 and below 50°. However, a maximum outlier can also be observed with approximately 90° of color. Moreover, boxplots from turbidity results (Figure 3a) and TSS results (Figure 3c), show the median closer to the middle, indicating that the water quality data distribution is symmetric or normal.

Table 7 shows a high correlation among parameters such as turbidity, color, and BOD with a correlation index ranging from 0.891 to 0.965. The high correlation between turbidity and TSS can also be highly linked to the fact that TSS is among the primary factors that are likely to affect the concentration of turbidity in water. Turbidity is a measure of how well light passes through liquid while TSS is a quantitative expression of particles in suspension [55].

**Figure 4:**
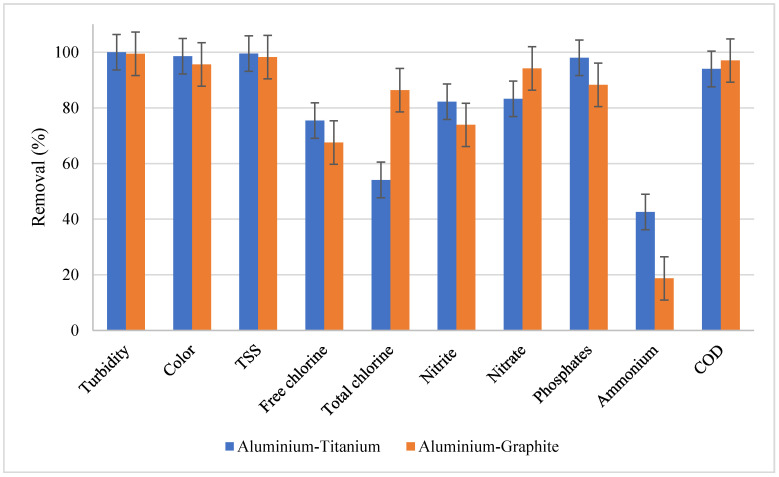
Removal efficiencies of the Al–Ti and Al–Gr combinations.

Generally, the average removal efficiencies from the studied electrode materials were successfully computed. From Figure 4, it can be observed that both Gr and Ti electrode materials connected to the Al electrode achieved high removal efficiency for parameters such as turbidity, color, TSS, and COD. The Al–Gr combination, however, faced more challenges on the ammonium removal with an average efficiency of 18.7%. However, the ammonium removal efficiency is also observed to be the lowest from the Al–Ti removal efficiencies. In general, the trend of removal efficiency is observed to be similar for both Gr and Ti electrodes connected to the Al electrode.

**Figure 5:**
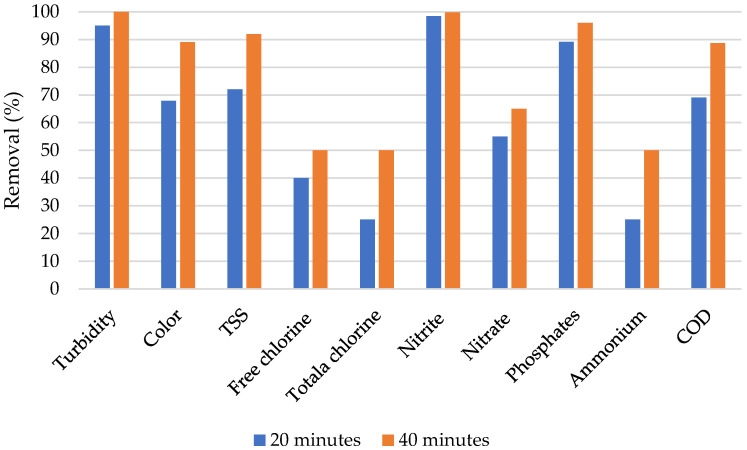
Removal efficiencies from the Al–Gr electrode combination.

From Figure 5, it can be observed that the removal performance from all the studied parameters increased with the increase in time. The results reveal that, under constant conditions, the performance of the Al–Gr electrode combination may be highly affected by the contact time. In the literature, Khandegar, Acharya, and Jain [56] investigated the effect of time on the removal efficiency of BOD and total dissolved solids (TDS) using EC methods, where a similar phenomenon (removal efficiency increase with time) was observed. In addition, from Figure 5, it can be observed that, with respect to the general effect of the contact time, each of the studied parameters had a different response to the treatment processes as the contact time increased. High removal efficiency can be observed from turbidity, color, nitrite, phosphates, and COD, with removal efficiency ranging from 72% to 98% after 20 min, as well as 88% to 100% after 40 min, while low removal efficiency can be observed from free and total chlorine as well as ammonium. Moreover, the results unveil that, free and total chlorine as well as ammonium may need higher contact time to achieve high removal efficiency using the Al–Gr electrode combination compared to the other parameters such as turbidity, color, nitrite, phosphates, and COD.

**Figure 6:**
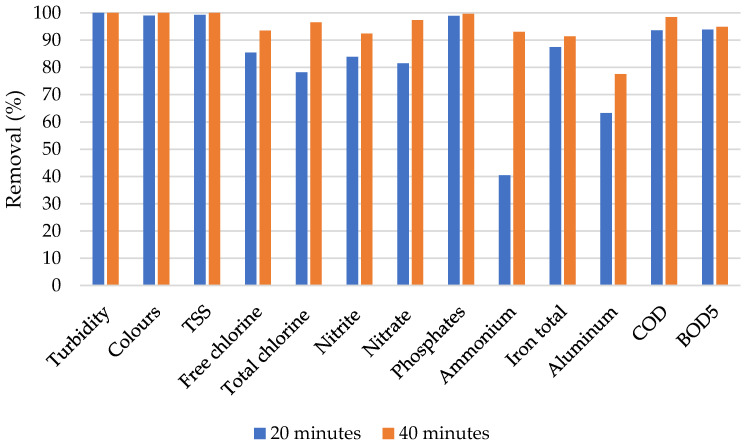
Removal efficiencies from the Al–Ti electrode combination.

From Figure 6, as observed from the Al–Gr electrode combination, a similar phenomenon (removal efficiency increase with time) can be seen from the Al–Ti electrode combination. High performance is also observed from turbidity, color, TSS, nitrite, phosphates, and COD, with removal efficiency ranging from 81% to 100% after 20 min as well as from 91% to 100% after 40 min. With the fact that as an extension to the list of parameters of interest was done when the wastewater was subjected to the Al–Ti electrode combination, the high removal efficiency from the treatment process can also be observed from total iron and BOD. Unlike the Al–Gr electrode combination, the Al–Ti electrode combination achieved 100% removal efficiency from turbidity after only 20 min of the contact time. The general performance in terms of removal efficiency indicates that the Al–Ti electrode combination outperformed the Al–Gr electrode combination.

### 3.2. Discharge Quality Compliance

The degree to which the treated effluent complies with some discharge requirements was also investigated in terms of percent compliance based on the World Health Organization (WHO) classification of surface freshwater quality for the maintenance of aquatic life [57]. The percent compliance was derived from average concentration values at the end of each experiment. From Table 8, it can be observed that all the parameters are within the permissible concentrations for discharge quality standards. In general, among the physicochemical parameters, BOD, COD, suspended solids, ammonia, nitrate, and nitrite as well as phosphates are regarded to be important parameters for discharge quality observance due to their significant impact on aquatic life and the environment in general [58]. In a case where a higher quality degree is required, such as meeting drinking water quality standards, the EC treatment systems have also been observed to be highly efficient when used as pre-treatment units [59], due to their ability to remove the majority of the suspended particles, as also observed in this study.

### 3.3. Operating Cost Analysis

Electrode and energy consumption are the main parameters that determine the operating cost for EC treatment technologies. Therefore, an increase in the two parameters also affects the total operating cost of an EC-based wastewater treatment technology. After conducting multiple experiments, the average operating cost for the Al–Gr combination was estimated to be 1.9 US$/m^3^ of purified water, while that of Al–Ti was estimated to be 3.57 US$/m^3^. The cost difference was highly influenced by the relatively high initial purchasing cost of the Ti electrode.

## 4. Conclusions

The potential of Gr and Ti electrodes in conjunction with the Al electrode for PSWW purification has been studied. The treatment approaches were investigated in terms of their general efficiency in removing physicochemical contaminants, the effect of the contact time as well as the operating cost associated with the treatment systems. The analysis results revealed that the Al–Gr electrode combination is highly efficient for the removal of turbidity, color, nitrite, phosphates, and COD, with removal efficiency ranging from 72% to 98% after 20 min, as well as 88% to 100% after 40 min. Similarly, the Al–Ti electrode combination was observed to be highly efficient in the removal of turbidity, color, TSS, nitrite, phosphates, and COD, with removal efficiency ranging from 81% to 100% after 20 min as well as from 91% to 100% after 40 min. The results further revealed that the contact time is among the crucial determinants of an EC treatment system for PSWW. In general, the performance of the electrode combinations in terms of the removal efficiency showed that the Al–Ti electrode combination has the potential to be more efficient in terms of the general treatment performance than the Al–Gr electrode combination when subjected to PSWW. However, the Al–Gr combination was observed to be more preferable in terms of operating costs mainly due to the inert properties of the Gr electrode. From this study, the optimum EC condition was observed from the turbidity removal as 100% efficiency was achieved within 20 min of the treatment process. In addition, all the studied parameters from both treatment approaches were observed to be in compliance with the discharge standards for the maintenance of aquatic life. In the future, it will be of great interest to extend the coverage of the contact time from below 20 min to above 40 min at smaller intervals.

## Figures and Tables

**Table 1:** General characteristics of raw wastewater (number of samples = 12).

Indicator	Min	Max	AM	SD	Unit
pH	6.5	7.5	6.998	0.410	–
Turbidity	43.6	647	218.883	200.652	FAU *
Color	319	4214	1999.167	1428.780	degree
TSS	75	1068	403.333	337.427	mg/L
Free chlorine	0	0.45	0.172	0.153	mg/L
Total chlorine	0	0.55	0.185	0.195	mg/L
Nitrite	0	0.181	0.092	0.058	mg/L
Nitrate	0	36.2	18.35	12.264	mg/L
Phosphates	2.67	5.79	4.838	1.018	mg/L
Ammonium	1.03	2.21	1.66	0.504	mg/L
COD	358	5009	2096.667	1628.197	mg/L
BOD5	139.6	1809	1124.633	524.713	mg/L
Total iron	0.19	1.33	0.673	0.497	mg/L
Manganese	0.26	0.49	0.387	0.093	mg/L
Nickel	3.73	8.61	5.902	1.931	mg/L
Chromium	0.56	2.16	1.095	0.582	mg/L
Aluminum	0.42	5.56	2.217	2.366	mg/L

* FAU = Formazin Attenuation Units.

**Table 2:** General technical specifications.

Parameter	Value	Unit
Initial water temperature	5–10	°C
Potential (voltage)	24	V
Average current density	5.5	A
Average power	132	W
Hydraulic retention time	40	min

**Table 3:** Interpretation of the correlation coefficients.

Range of Correlation Coefficient	Strength of Relationship
0–0.29	Weak
0.3–0.49	Moderate
0.5–0.69	Strong
0.7–1	Very strong

**Table 4:** General physicochemical results from the Al–Gr treatment approach (number of samples = 12).

Indicator	Min	Max	AM	SD	Unit
pH	4.15	6.49	6.042	0.786	–
Turbidity	0	4.44	1.252	1.695	FAU
Color	10	239	88	75.633	°
TSS	0	15	7.167	5.398	mg/L
Free chlorine	0.01	0.11	0.062	0.037	mg/L
Total chlorine	0.01	0.22	0.103	0.082	mg/L
Nitrite	0.001	0.088	0.024	0.031	mg/L
Nitrate	0	3.9	1.067	1.297	mg/L
Phosphates	0.08	1.64	0.568	0.518	mg/L
Ammonium	0.04	2.31	1.352	0.925	mg/L
COD	0	177	62.75	57.508	mg/L

**Table 5:** Correlation of some physicochemical parameters from the Al–Gr combination.

Indicator	Turbidity	Color	TSS	Phosphates	Ammonium	COD
Turbidity	1	–	–	–	–	–
Color	0.909	1	–	–	–	–
TSS	0.907	0.890	1	–	–	–
Phosphates	−0.054	0.286	0.247	1	–	–
Ammonium	0.527	0.556	0.429	0.348	1	–
COD	0.947	0.990	0.922	0.185	0.491	1

**Table 6:** Physicochemical results from the Al–Ti treatment system.

Indicator	Min	Max	AM	SD	Unit
pH	6.80	7.50	7.18	0.25	–
Turbidity	0.00	0.00	0.00	0.00	FAU
Color	0.00	59.00	29.00	18.10	°
TSS	0.00	5.00	2.00	1.63	mg/L
Free chlorine	0.01	0.07	0.05	0.02	mg/L
Total chlorine	0.02	0.24	0.08	0.08	mg/L
Nitrite	0.00	0.04	0.02	0.01	mg/L
Nitrate	0.40	6.90	3.07	2.45	mg/L
Phosphates	0.02	0.19	0.10	0.06	mg/L
Ammonium	0.00	2.13	0.76	0.96	mg/L
COD	4.80	412.00	120.33	170.23	mg/L
BOD	4.68	31.20	15.95	9.44	mg/L
Total iron	0.02	0.16	0.05	0.05	mg/L
Manganese	0.11	0.29	0.18	0.07	mg/L
Nickel	1.60	3.58	2.45	0.83	mg/L
Chromium	0.10	0.46	0.38	0.13	mg/L
Aluminum	0.11	1.60	0.65	0.68	mg/L

**Table 7:** Correlation of some physicochemical parameters from the Al–Ti combination.

Indicator	Turbidity	Color	TSS	Nitrate	Phosphates	Ammonium	BOD
Turbidity	1	–	–	–	–	–	–
Color	0.965	1	–	–	–	–	–
TSS	0.891	0.896	1	–	–	–	–
Nitrate	0.304	0.246	0.133	1	–	–	–
Phosphates	0.611	0.587	0.298	0.809	1	–	–
Ammonium	0.147	0.090	−0.029	0.891	0.765	1	–
BOD	0.899	0.891	0.957	0.213	0.341	−0.063	1

**Table 8:** Percent compliance based on surface freshwater quality guideline.

Indicator	Guideline	Al–Gr	Al–Ti
рН	(6–9)	100	100
Turbidity	50	100	100
Color	50	58	100
TSS	50	96	100
Nitrate plus nitrite	100	99.3	99.2
Phosphates	2	89	99
Ammonium	2.5	98.4	95.2
COD	125	61.6	95.6
BOD	30	–	86.8
Manganese	50	–	99.63667
Nickel	15	–	83.64444
Chromium	5	–	92.4
Aluminum	5	–	87.06667
